# Gallbladder perforation in photon counting CT: A case report

**DOI:** 10.1016/j.radcr.2025.05.015

**Published:** 2025-05-30

**Authors:** Wenjing Li, Song Luo

**Affiliations:** a Department of Radiology, Geriatric Hospital of Nanjing Medical University, Nanjing, China; b Department of Radiology, Jiangsu Province Official Hospital, Nanjing, Jiangsu, China

**Keywords:** Photon-counting CT, Common bile duct stones, Gallbladder perforation, Acute cholecystitis, Biliary obstruction

## Abstract

A 74-year-old male presented to the emergency department with a 5-day history of abdominal pain and fever. Laboratory tests suggested infection and biliary obstruction. Contrast photon-counting computed tomography (PCCT) imaging revealed gallstones, acute cholecystitis, gallbladder perforation, and dilation of the upper segment of the common bile duct, with a suspicion of a potential common bile duct stone (CBDS) that was not visualized. When the energy level was adjusted to 120 keV during postprocessing, a tiny, high-density nodule in the common bile duct became visible. It was further enhanced using a pseudo-colored gallstone template. Surgical exploration confirmed the presence of CBDS. CBDS is often associated with complications such as biliary obstruction, cholangitis, and pancreatitis. In this case, identifying a tiny CBDS after initial contrast imaging suggested that CBDS may have been the underlying cause of the gallbladder perforation. PCCT’s multi-energy capabilities played a crucial role in improving diagnostic accuracy and provided clinical insights into the possible cause of the perforation. This case demonstrates the importance of PCCT in diagnosing tiny CBDS, which may change the clinical understanding of gallbladder perforation etiology.

## Introduction

Common bile duct stones (CBDS) are a recognized cause of significant clinical morbidity due to their potential to induce biliary obstruction, acute cholecystitis, cholangitis, and pancreatitis. Early and accurate diagnosis of CBDS is essential for determining effective therapeutic strategies and preventing further complications. Traditional imaging techniques, such as ultrasound and conventional computed tomography (CT), often face challenges in detecting tiny common bile duct stones (CBDS), particularly in complex biliary conditions where other abnormalities, such as gallbladder perforation may obscure findings. Photon-counting computed tomography (PCCT) is an advanced high-resolution imaging technology that leverages multi-energy reconstruction to improve tissue and stone differentiation. PCCT is an innovative imaging technique that utilizes photon-counting detectors to directly measure each incoming photon, capturing multienergy data simultaneously in a single scan. Compared to conventional energy-integrating CT, PCCT provides higher spatial resolution and improved contrast-to-noise ratio, facilitating more precise tissue characterization and enhanced differentiation between calcified structures such as tiny bile duct stones and surrounding soft tissues. The multi-energy reconstruction capabilities of PCCT allow clinicians to optimize image quality by selecting specific keV levels postacquisition, significantly enhancing diagnostic accuracy. In this case, PCCT successfully identified a tiny CBDS that was inconspicuous on the initial contrast imaging. Detection of this stone during the adjustment of different keV levels suggested the possibility that CBDS might be the primary cause of gallbladder perforation. This conclusion could not have been drawn solely from the initial imaging. This case report highlights the critical role of PCCT in detecting tiny CBDS and its potential utility in elucidating the etiology of gallbladder perforation.

## Case report

A 74-year-old male presented to the emergency department with a 5-day history of persistent right upper quadrant abdominal pain and fever, with a peak temperature of 39°C. The patient had a history of gallstones for several years and reported similar mild episodes in the past but had not received prior surgical intervention. He denied alcohol use, smoking, or any previous episodes of jaundice or pancreatitis.

His medical history included hypertension for 15 years, managed with daily amlodipine, and cerebral infarction 10 years ago, which was managed conservatively. Two years earlier, he had undergone upper and lower thoracoscopic pulmonary lobectomy and pleural adhesiolysis under general anesthesia. Pathology revealed 2 distinct foci of invasive pulmonary adenocarcinoma: a moderately differentiated acinar-predominant lesion (1.8 × 1.0 × 0.8 cm, PL0) in the right lower lobe and a poorly differentiated high-grade adenocarcinoma (3.7 × 2.5 × 2.0 cm, PL1 with airway spread) in the right upper lobe. Genetic testing showed EGFR exon 20 insertion mutation with p.S768I and p.V774M variants. The patient received 4 cycles of chemotherapy with pemetrexed and carboplatin, with dose adjustments due to poor tolerance, and follow-up imaging showed stable disease. There was no family history of hepatobiliary, cardiovascular, or oncologic conditions.

Physical examination revealed abdominal distension, right upper quadrant tenderness, and a positive Murphy’s sign. Laboratory tests showed leukocytosis (WBC 13.4 × 10⁹/L), neutrophilia (7.45 × 10⁹/L, 84.6%), lymphopenia (0.76 × 10⁹/L), mild anemia (RBC 4.15 × 10¹²/L; hemoglobin 131 g/L), and thrombocytopenia (platelets 122 × 10⁹/L). Inflammatory markers, including high-sensitivity C-reactive protein (123.35 mg/L) and procalcitonin (5.360 ng/mL), were significantly elevated. Liver function tests revealed elevated ALT (65 U/L), AST (86 U/L), GGT (241 U/L), ALP (153 U/L), and total/direct bilirubin levels (total 47.20 µmol/L, direct 14.2 µmol/L), suggesting biliary obstruction. Pancreatic enzymes were mildly elevated (amylase 105 U/L, lipase 45.9 U/L). Electrolyte analysis showed hyponatremia (Na⁺ 128 mmol/L) and mild hyperkalemia (K⁺ 4.95 mmol/L), while renal function was mildly impaired (creatinine 110.7 µmol/L, urea 8.2 mmol/L). Coagulation studies revealed prolonged fibrinogen (6.07 g/L, ref: 2.38-4.98), elevated D-dimer (1.58 mg/L, ref: 0-0.234), but standard prothrombin time (13.4 s), INR (1.25), aPTT (29.5 s), and thrombin time (12.3 s), suggesting hypercoagulable status secondary to inflammation or sepsis.

**Fig. 1:**
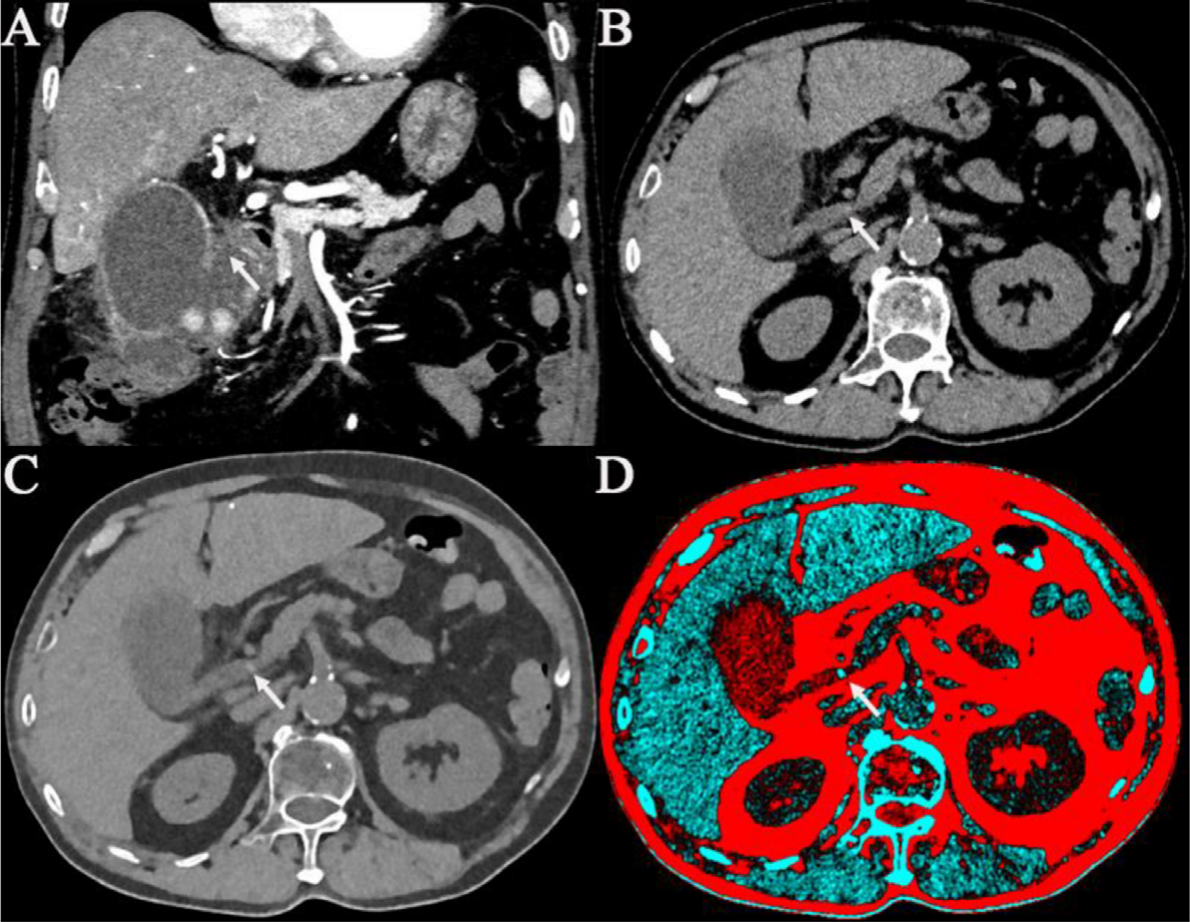
Photon-counting CT (PCCT) imaging of the abdomen shows gallstones, upper common bile duct dilation, and gallbladder perforation. Contrast-enhanced CT images in the coronal plane demonstrate hyperdense nodules, fluid accumulation around the gallbladder and bile duct walls, which appear thickened with enhancement. Specifically, the PCCT imaging revealed a localized defect in the gallbladder wall, accompanied by pericholecystic fluid collection, indicating bile leakage secondary to gallbladder perforation (A). The 60 keV PCCT image (B) reveals dilation of the common bile duct and suspected common bile duct (arrows). The 120 keV PCCT image (C) shows a more clearly defined common bile duct. A dedicated pseudo-color template for gallstones (D) enhances its visualization (arrows).

The patient underwent initial contrast abdominal PCCT imaging with a Siemens NAEOTOM Alpha photon-counting CT scanner using standard abdomen protocol parameters (slice thickness: 0.4 mm, collimation: 144 × 0.4 mm, tube voltage: 120 kVp, automated exposure control). Specifically, the PCCT imaging revealed a localized defect in the gallbladder wall, accompanied by pericholecystic fluid collection, indicating bile leakage secondary to gallbladder perforation (Fig. 1(A)). Initial images at 60 keV Fig. 1(B) showed gallbladder perforation and dilation of the common bile duct, though the common bile duct stone (CBDS) was not visible. During the postprocessing stage, multi-energy analysis software was utilized to adjust the reconstructed image energy levels incrementally. At the optimized energy level of 120 keV (Fig. 1(C)), the tiny high-density nodule within the common bile duct became distinctly apparent. A pseudo-colored gallstone-specific template Fig. 1(D) was subsequently applied to enhance the visualization of the stone further, clearly delineating its presence and location, significantly aiding in surgical planning and intervention. Due to the patient’s severe clinical condition at emergency presentation, characterized by clear imaging evidence of gallbladder perforation and biliary dilation and high clinical suspicion of common bile duct stones, preoperative magnetic resonance cholangiopancreatography (MRCP) was not performed. The decision was made to expedite urgent surgical intervention.

Based on these findings, the patient underwent an urgent laparoscopic cholecystectomy with COOK stone removal and basket extraction of the common bile duct stones. Intraoperative cholangioscopy confirmed the presence of a hard black stone in the common bile duct, along with acute purulent cholecystitis, gallbladder perforation, and cholangitis. Intraoperative findings definitively confirmed that the common bile duct stone (CBDS) caused biliary obstruction, subsequently leading to acute cholecystitis and gallbladder perforation, clearly establishing the CBDS as the direct cause of the patient’s clinical symptoms.. The patient recovered well after surgery and was discharged from the hospital 2 weeks later, with follow-up planned for an outpatient setting.

## Discussion

CBDS present a substantial diagnostic and therapeutic challenge, as they can lead to severe complications such as biliary obstruction., cholangitis, and pancreatitis. Early detection of CBDS is critical for effective management of these complications. In this case, the CT imaging revealed gallbladder stones, acute cholecystitis, gallbladder perforation, and dilation of the upper common bile duct, but the CBDS was not visualized. After postprocessing adjustments, including applying a 120 keV energy setting, the CBDS became evident.

Indentifying a tiny CBDS on PCCT raises critical questions about the underlying cause of gallbladder perforation. Obstruction caused by CBDS can elevate pressure within the biliary system, potentially leading to ischemia and weakening of the gallbladder wall, ultimately resulting in perforation. The observed bile duct dilation above the stone supports the hypothesis that CBDS was the primary driver of the obstruction, contributing to the perforation.

While urgent surgical intervention was necessary in this case due to the gallbladder perforation, PCCT played a pivotal role in uncovering the underlying cause. In clinical practice, PCCT protocols typically involve acquiring high-resolution, multi-energy data with precise collimation and thin-slice reconstructions, enabling detailed visualization of tiny structures within the biliary system. The ability to retrospectively adjust imaging energy levels and apply pseudo-color templates for specific tissues or structures constitutes a significant diagnostic advantage. This capability proved critical in our case, as detecting the CBDS at 120 keV prompted immediate and targeted surgical intervention, potentially reducing complications associated with delayed or missed diagnosis. Additionally, the ability to visualize the stones at 120 keV during postprocessing and the use of pseudo-color templates highlights the unique diagnostic advantages of PCCT in managing complex biliary tract conditions. The improved visibility of the ductal stone at the higher energy level (120 keV) can be attributed to its high calcium content. Bile duct stones typically contain cholesterol or calcium bilirubin, which include dense calcium components. These calcium-rich stones exhibit more significant attenuation differences at higher energy levels, resulting in enhanced contrast between stones, biliary fluid, and surrounding soft tissues, thus facilitating the detection of tiny stones. Compared to gallbladder stones, the ductal stone was less conspicuously visualized in initial lower-energy images. This observation may be attributed to differences in composition and smaller size. Specifically, common bile duct stones often contain relatively less calcium than gallbladder stones, resulting in lower overall density and, consequently, poorer conspicuity at lower keV levels. PCCT’s ability to differentiate between stones and surrounding soft tissues in the bile duct could alter preoperative planning and intraoperative management. Specifically, detecting a small CBDS may prompt more careful exploration of the common bile duct during surgery, potentially minimizing the risk of residual stones and subsequent cholangitis. PCCT may serve as a valuable tool in cases of ambiguous biliary pathology, guiding clinicians toward more targeted therapeutic interventions.

PCCT provides high-resolution images and multi-energy data acquisition in a single scan [1], greatly enhancing diagnostic precision and boosting clinical confidence. PCCT’s high-resolution and multi-energy reconstruction capabilities enhance tissue differentiation and allow more precise visualization of tiny stones [2], especially in complex anatomical areas such as the common bile duct. Pseudo-color imaging further enhances diagnostic accuracy by highlighting differences between stones and surrounding tissues.

This case highlights the clinical significance of PCCT in detecting tiny CBDS and potentially elucidating the cause of gallbladder perforation. Traditional imaging methods may miss tiny stones, leading to insufficient diagnoses and suboptimal treatment plans. In this context, ‘traditional imaging methods’ refer to conventional ultrasound, standard computed tomography (CT), and magnetic resonance cholangiopancreatography (MRCP). PCCT, with its superior resolution and tissue differentiation capabilities, provides a more comprehensive assessment of the biliary system, improving diagnostic confidence and guiding more effective clinical management.

In conclusion, PCCT represents a significant advancement in diagnosing and managing biliary diseases. In this case, PCCT played a crucial role in identifying a tiny CBDS that was likely the underlying cause of gallbladder perforation. This diagnosis could have been missed with conventional imaging methods. The ability of PCCT to visualize tiny stones and provide detailed anatomical information is essential for improving patient outcomes in cases of complex biliary conditions.

## Patient consent

The authors confirm that written informed consent was obtained from the patient for the publication of this case report and any accompanying images. The patient has been made aware that all identifying information will be kept confidential, and every effort has been made to ensure their privacy. This consent documentation is retained by the authors and is available upon request by the journal.

## Data sharing statement

In compliance with patient confidentiality requirements, our manuscript ensures that all personal data is protected. No identifiable information, such as patient names, hospital ID numbers, or other unique identifiers, appears in any images, illustrations, or filenames.
